# Neuronal and glial networks interact with traumatic brain injury to modulate cognition in ABCD study

**DOI:** 10.1038/s41540-026-00681-8

**Published:** 2026-03-13

**Authors:** Michael Cheng, Melody Mao, Wenjing Meng, Joanna Jacobus, Emily A. Troyer, Everett L. Delfel, Emily L. Dennis, Elisabeth A. Wilde, Tracy Abildskov, Nicola L. de Souza, Florin Vaida, Jeffrey E. Max, Xia Yang

**Affiliations:** 1 Department of Integrative Biology & Physiology, University of California, Los Angeles, Los Angeles, CA USA; 2 Bioinformatics Interdepartmental Program, University of California, Los Angeles, Los Angeles, CA USA; 3 Division of Biostatistics, School of Public Health, University of California, San Diego, San Diego, CA USA; 4 Department of Psychiatry, University of California, San Diego, San Diego, CA USA; 5 Joint Doctoral Program in Clinical Psychology, San Diego State University / University of California, San Diego, San Diego, CA USA; 6 Department of Neurology, University of Utah School of Medicine, Salt Lake City, UT USA; 7 George E. Wahlen Veterans Affairs Medical Center, Salt Lake City, UT USA; 8 Molecular, Cellular and Integrative Physiology Interdepartmental Program, University of California, Los Angeles, Los Angeles, CA USA; 9 Molecular Biology Institute, University of California, Los Angeles, Los Angeles, CA USA; 10 Brain Research Institute, University of California, Los Angeles, Los Angeles, CA USA; 11 Institute for Quantitative and Computational Biosciences, University of California, Los Angeles, Los Angeles, CA USA; 12 Department of Molecular and Medical Pharmacology, University of California, Los Angeles, Los Angeles, CA USA

**Keywords:** Genetics, Neurology, Neuroscience

## Abstract

Mild traumatic brain injury (mTBI) disproportionately affects children and adolescents and has been associated with poorer neurocognitive performance, but the biological mechanisms driving symptom variability and severity remain understudied. In accordance with the omnigenic disease model, we integrated gene-by-mTBI interaction genome-wide association studies on neurocognition from the Adolescent Brain Cognitive Development (ABCD) cohort with single-cell RNA sequencing gene regulatory networks to elucidate the cell type-specific key regulators and molecular mechanisms governing neurocognitive outcome of mTBI, specifically learning and memory performance. Our analysis revealed distinct network regulators in neuronal and glial cell types across hippocampal and cortical brain regions to orchestrate key neurodevelopmental pathways. Examples include *APP* for synaptic signaling in excitatory neurons, *COX5A* for mitochondrial function in inhibitory neurons, *MOG* for myelination in oligodendrocytes in the hippocampus; *GRM7* for synaptic signaling in excitatory neurons, *SV2A* for synaptic signaling in inhibitory neurons, and *MOG* for myelination in oligodendrocytes in the cortex. These mechanisms also associate with learning and memory through pathway-based polygenic risk score modeling in ABCD. Our findings provide brain region- and cell type-specific insights into the complex regulatory network landscape of mTBI pathology and potential therapeutic candidates at the pathway and network levels.

## Introduction

Traumatic brain injury is a global public health concern, with an estimated 69 million injuries occurring worldwide each year and 1.4 million reported annually in the United States alone^1,2^. Mild traumatic brain injury (mTBI), commonly known as concussion, is the most prevalent form of TBI, accounting for 70–90% of all cases^3^. Although “mild” suggests favorable prognosis, the effects of mTBI vary between individuals and can have lasting impacts on neural and psychological development^4–7^. With one-third of all mild TBIs occurring in children under the age of 15, accurate prognostication of injury outcomes in children and adolescents is necessary^8^. However, current mTBI prognostic models fail to capture the wide variation in patient outcomes^5,7^, which is likely driven by the poorly understood genetic mechanisms that interact with mTBI to affect acute and chronic neurocognitive outcomes differently between individuals.

The broad pathological impact of mTBI on multiple brain regions and cell types, coupled with the numerous genetic variants previously uncovered for neurocognition, implicates an omnigenic disease model that may explain the variation in mTBI outcomes^9–13^. The omnigenic disease model explains that all genes in a system operate in complex regulatory networks to affect disease, with a few core network genes with strong regulatory effects and many more peripheral genes with weaker effects that collectively contribute more to the trait variability^14,15^. Thus, a genome-wide study of gene networks across brain regions and cell types affecting neurocognitive variability in mTBI has the potential to provide holistic insights into the underlying mechanisms driving outcome severity.

However, with limited mTBI patient cohort sizes between 40 and 100, recent studies resort to targeted genetic studies because of the lack of statistical power to conduct unbiased genome-wide association studies (GWAS). These studies have proposed candidate genetic variants in core genes in neuronal repair, inflammation, and mitochondrial dysfunction, such as *APOE*, *DRD2*, *COMT*, *BDNF*, and *BCL2*^16–28^. As core genes explain less of the cumulative genetic variation in disease compared to peripheral gene networks, employing systems biology approaches to genome-wide interactions with mTBI and their clinical manifestations remains an ongoing effort.

Current systems biology studies on pediatric patients with mTBI, compared to orthopedic injury (OI), have revealed marginal and aggregate associations of neuronal and inflammatory pathways with child behavioral adjustment and executive function, specifically in mTBI patients^24,29^. These studies explore a few predefined gene sets containing genes related to TBI in cohorts with sample sizes less than 100 patients, which limits the possibility of discovering novel pathways. Additionally, the focus on general gene sets and pathways overlooks the tissue and cell type-specific activity of these genes, which control mTBI pathology through complex regulatory networks. While emerging single-cell transcriptomic studies on mTBI mouse models have demonstrated the cell type-specific responses in mTBI^9–12^, there remains a need to uncover whether these networks interact with genetic risks to determine TBI outcomes. Moreover, there is a lack of data- and network-driven studies on neurocognitive areas in the context of mTBI, like learning and memory performance, despite the well-known association between mTBI and cognitive performance.

**Fig. 1:**
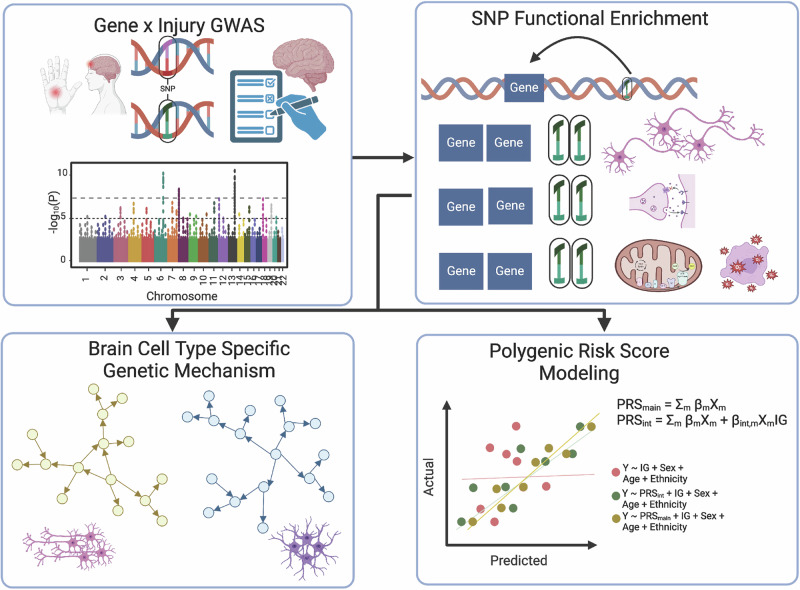
Study overview of the multiomics systems biology analysis. First, a gene-by-environment (mTBI) GWAS on learning and memory composite scores was performed, followed by SNP to gene mapping and biological pathway enrichment of GWAS signals. These pathways are overlaid onto cell type gene regulatory networks for mechanistic study and used for polygenic risk analysis for clinical modeling. Created in BioRender. Yang, X. (2026) https://BioRender.com/orw6tm5.

The Adolescent Brain Cognitive Development (ABCD) cohort provides an opportunity to conduct systems biology studies on how mTBI interacts with genetic risks to affect molecular networks underlying neurological outcome^30,31^. With over 11,000 children enrolled in a ten-year longitudinal study that measures diverse clinical variables, brain scans, and genetic data, ABCD contains the largest available mTBI (*n* = 423) and OI (*n* = 1469) populations to perform gene-by-mTBI interaction analyses, which can be compared to genetic loci associated with a broad range of neurological traits and diseases from existing GWAS as orthogonal validation. We hypothesize that genetic variants that interact with mTBI follow an omnigenic model and act through cell type-specific biological networks and pathways to modify cognitive outcome. We aim to test this hypothesis by integrating the full spectrum of gene-by-mTBI interaction signals from ABCD with biological pathways and gene network models from brain single-cell RNA sequencing (scRNA-seq) data to uncover brain region- and cell type-specific gene regulatory networks that interact with mTBI to affect cognitive outcomes. As depicted in the schematic overview of study design in Fig. 1, we first conduct a gene-by-mTBI interaction GWAS on learning and memory performance, captured as neurocognitive principal component 3 (NPC3) derived from the NIH Toolbox^32^. Using the full distribution of GWAS summary statistics, we identify enriched biological pathways. These pathways are then overlaid onto brain region- and cell type-specific gene regulatory networks to elucidate key networks that mediate gene-by-mTBI interaction. Lastly, we explore the clinical utility of pathway-based polygenic risk scores (PRS) to predict neurocognitive outcomes.

## Results

### Additive GWAS on learning and memory in ABCD recapitulates existing memory GWAS

Before exploring gene-by-mTBI interaction on NPC3 (learning and memory), as a positive control, we first assessed if a standard additive NPC3 GWAS without an interaction term concurs with previous learning and memory GWAS. The NPC3 additive GWAS included SNPs, injury type (mTBI vs OI), age, sex, and the top 20 genetic principal components representing the overall genetic architecture for population ancestry differences (Supplementary Fig. 1a and Supplementary Data 1). We observed no inflation of population structure or biases in our GWAS (genomic control inflation factor λGC = 1) with a slight deflation for lower *p* values compared to the expected in the quantile-quantile (QQ) plot due to our modest sample size (Supplementary Fig. 1b)^33^. The correlations of SNP effect size estimates from our ABCD GWAS across all SNPs with previous working memory GWAS^34–38^ with larger population sizes (450,000–500,000 individuals) were significantly greater than those with unrelated phenotypes (fasting blood glucose^39^, grip strength^40^, low density lipoprotein cholesterol^41^, ascending aortic diameter^42^, hemoglobin levels^43^, alkaline phosphatase^44^, serum albumin levels^45^, bone mineral density^46^, triglyceride levels^47^) from the GWAS catalog (Supplementary Fig. 1c, *p* < 0.001 by Wilcoxon rank-sum test; Supplementary Data 2)^34–38^. These results support that the ABCD cohort recapitulates the main genetic effect of a similar cognitive memory trait and provide confidence for conducting gene-by-mTBI interaction analyses.

### Gene-by-mTBI interaction GWAS on learning and memory NPC3 reveals SNPs relevant to cognitive traits

**Fig. 2:**
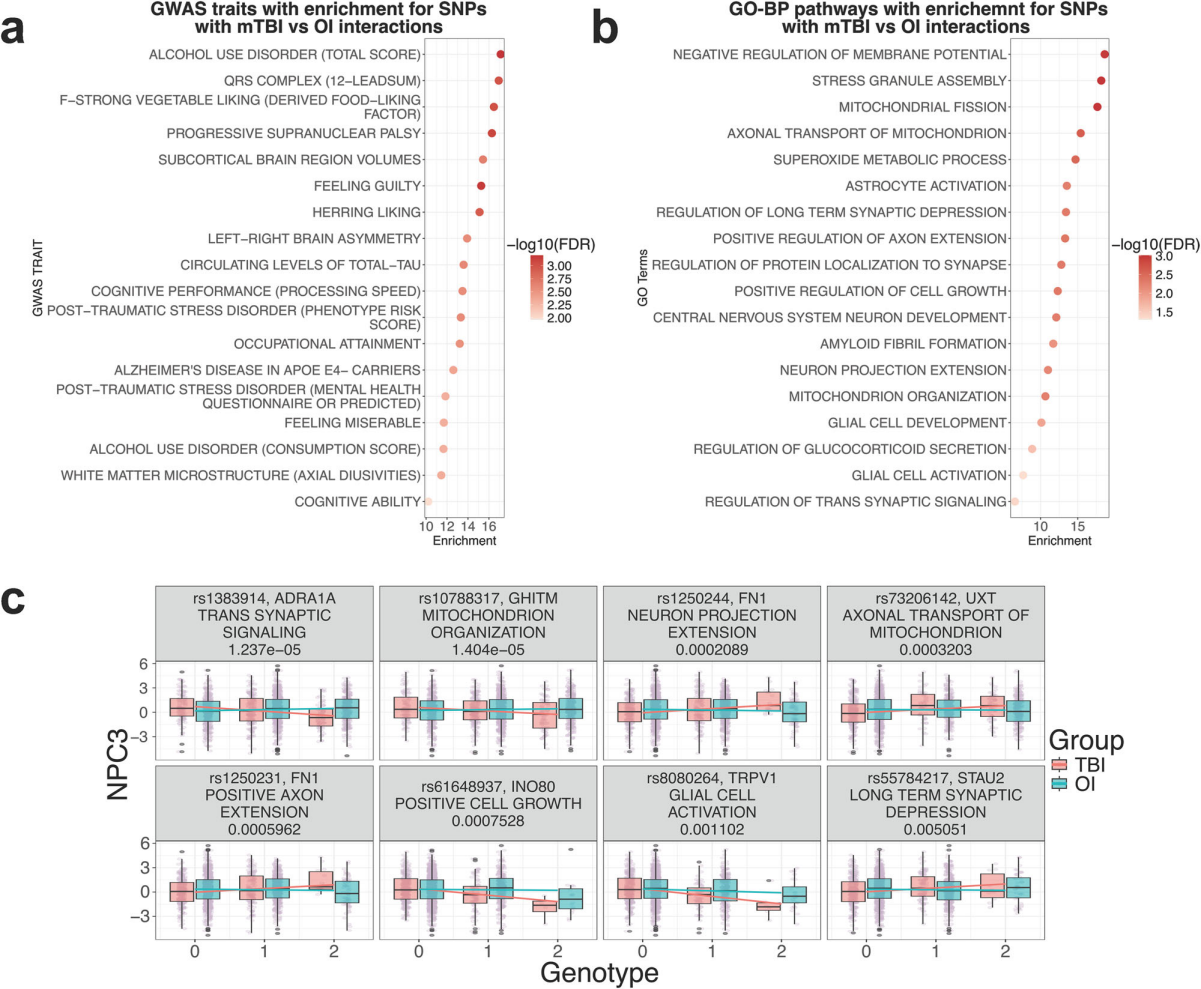
SNP-by-mTBI interaction GWAS interpretation. **a** Significant top traits in the GWAS catalog whose candidate genes are enriched for stronger SNPxInjury association in ABCD. Color indicates FDR after BH correction. **b** Significant top pathways in the Gene Ontology Biological Pathways whose genes are enriched for stronger SNPxInjury association in ABCD. Color indicates FDR after BH correction. **c** Learning and memory score distribution for top SNPs in select GO pathways in Fig. 2D. Color corresponds to injury group (mTBI in red; OI in cyan).

To understand the presence of gene-by-mTBI interactions in association with learning, we added an interaction term between the SNP and injury type to account for interactive effects between mTBI (vs OI) and genetic variants into the above additive GWAS, adjusting for the same covariates (Supplementary Fig. 2a and Supplementary Data 3). The SNPs from the interaction term implicates genetic variants with differential effects on cognition between mTBI and OI. The use of OI as a reference is common in mTBI studies as it enables the control of factors that predispose children to accidents and psychological trauma associated with an injury and its treatments^48^. Like the additive model, the interaction GWAS is well-calibrated (λGC = 0.981) with a slight deflation for lower *p*-values from the expected in the QQ plot, likely due to conservative genetic PC overcorrection and/or the modest sample size (Supplementary Fig. 2b)^33,49^. To evaluate whether the SNP-by-mTBI interactions revealed from ABCD are biologically relevant, we ran Marker Set Enrichment Analysis (MSEA) using candidate gene sets from 2891 GWAS traits in the GWAS catalog and found that 60 traits were significantly enriched for the NPC3 interaction SNPs after multiple testing correction. Among the 60 GWAS traits, 34 are related to mTBI, cognition and intelligence, such as cognitive ability, post-traumatic stress disorder, occupational attainment, household income, and dyslexia (Fig. 2a and Supplementary Data 4). Furthermore, the SNPs were significantly more enriched in TBI-related traits than unrelated traits, independently selected from the GWAS catalog by two neuroscientists (Wilcoxon rank-sum test *p* = 0.03 and *p* = 0.002, respectively, Supplementary Fig. 2b and Supplementary Data 5, details in Methods). These results support that the genetic association signals from our SNP-by-mTBI interaction term recapitulate neurological pathways involved in cognitive traits known to be associated with TBI.

### Biological pathways informed by SNP-by-mTBI interaction GWAS on learning and memory NPC3

To interpret the functional implications of SNP-by-mTBI interaction SNPs using biological pathways, we performed the Marker Set Enrichment Analysis (MSEA) analysis on 5281 Gene Ontology biological pathways (GO-BP). We observed significant enrichment of our SNP-by-mTBI interaction GWAS SNPs in 137 pathways at a false discovery rate (FDR) <5%, many of which were related to known TBI pathological mechanisms (Table 1 and Supplementary Data 6). Pathways with the highest enrichments fell into broad categories of mitochondrial organization and metabolism, cytoskeletal organization and protein transport, neuronal development, synaptic signaling, and glial cell activation (Fig. 2b). These results were also observed when repeating the analysis in a SNP-by-mTBI interaction GWAS on NPC3 in the European population in ABCD, supporting the validity and ancestry-agnostic role of these pathways in interacting with mTBI to affect neurocognition (Supplementary Data 7 and Supplementary Fig. 2d). To visualize the interaction effect, we plotted the SNPs with the strongest interaction effects from the interaction GWAS within the top significant pathways. The genotypes of these SNPs indeed show differential association with learning/memory NPC3 scores between the ABCD mTBI and OI groups (Fig. 2c).

**Table 1:** MSEA analysis of GO-BP pathways for enrichment of SNP-by-mTBI GWAS signals

Pathway	FDR	MSEA enrichment score	Top genes	Top SNPs in SNP-by-mTBI interaction term	-log10 (GWAS P)
Mitochondrial fission	9.72E-04	17.65	MAPT; AURKA; DDHD2; PINK1; FIS1	rs17763658; rs59173918; rs72644707; rs12401770; rs150814238	3.77; 3.65; 3.54; 3.00; 2.80
Stress granule assembly	1.01E-03	18.17	MAPT; PRKAA2; BICD1; LSM14A; PUM2	rs17763658; rs4568857; rs144166034; rs570643; rs74333499	3.77; 2.65; 2.53; 2.14; 2.11
Negative regulation of membrane potential	1.08E-03	18.63	MAPT; TRPV1; PMAIP1; ARL6IP5; BAX	rs17763658; rs9905037; rs58697140; rs4407408; rs12460533	3.77; 3.62; 2.29; 2.26; 2.16
Axonal transport of mitochondrion	2.46E-03	15.40	MAPT; TRAK1; OPA1; AGBL4; AGTPBP1	rs17763658; rs9861303; rs6771174; rs320028; rs79634858	3.77; 2.75; 2.60; 2.25; 2.17
Superoxide metabolic process	3.03E-03	14.71	MAPT; IMMP2L; NOX1; CYBA; ACP5	rs17763658; rs17158597; rs7128; rs13334987; rs4804617	3.77; 3.25; 3.08; 3.06; 2.85
Positive regulation of axon extension	4.51E-03	13.31	TRPC5; MAPT; FN1; NTN1; DSCAM	rs3027784; rs17763658; rs1250244; rs62068228; rs7277154	3.77; 3.77; 3.68; 3.26; 2.78
Positive regulation of cell growth	4.61E-03	12.32	SLC44A4; IST1; TRPC5; MAPT; FN1	rs146535109; rs79169855; rs3027784; rs17763658; rs1250244	4.69; 3.88; 3.77; 3.77; 3.68
Regulation of protein localization to synapse	4.74E-03	12.80	MAPT; ARHGAP44; DVL1; GHSR; WNT7A	rs17763658; rs12950148; rs2296475; rs595161; rs11128663	3.77; 3.39; 2.87; 2.83; 2.72
Regulation of long-term synaptic depression	4.98E-03	13.42	MAPT; GRID2; ADCY8; STAU2; FMR1	rs17763658; rs17274136; rs191736; rs55784217; rs25726	3.77; 2.57; 2.54; 2.30; 2.20
Central nervous system neuron development	5.20E-03	12.12	ZMIZ1; MAPT; NDNF; DRD1; NIN	rs704016; rs17763658; rs17051286; rs267373; rs2984270	4.87; 3.77; 3.47; 3.41; 3.41
Astrocyte activation	5.41E-03	13.54	MAPT; IL1B; IFNGR1; LDLR; MT1X	rs17763658; rs1143629; rs4896242; rs379309; rs12934069	3.77; 2.52; 2.37; 2.26; 2.20
Mitochondrion organization	5.44E-03	10.65	GHITM; HSPA1A; HSPA1L; ERBB4; MAN2A1	rs10788317; rs146535109; rs146535109; rs2165098; rs2033463	4.85; 4.69; 4.69; 4.15; 3.77
Neuron projection extension	6.75E-03	11.00	TRPC5; MAPT; FN1; AURKA; CPNE5	rs3027784; rs17763658; rs1250244; rs59173918; rs6920224	3.77; 3.77; 3.68; 3.65; 3.62
Amyloid fibril formation	8.83E-03	11.71	MAPT; GSN; SIAH2; IAPP; RIPK3	rs17763658; rs2304392; rs73869296; rs28446944; rs17256902	3.77; 3.12; 2.92; 2.83; 2.65
Glial cell development	1.31E-02	10.11	MAPT; ASPA; SOX11; DRD1; CDK6	rs17763658; rs9905037; rs76251724; rs267373; rs11974095	3.77; 3.62; 3.42; 3.41; 3.23
Regulation of glucocorticoid secretion	2.42E-02	8.87	CRHR1; TSPO; GAL; POMC; GALR1	rs7222658; rs138952; rs59226850; rs3754863; rs2849291	3.99; 2.67; 2.27; 1.77; 1.68
Regulation of trans synaptic signaling	4.18E-02	6.55	DYSF; ADRA1A; PLCB1; ERC2; SYN1	rs4852258; rs1383914; rs75234999; rs56242225; rs4824626	5.11; 4.91; 4.41; 4.36; 3.99
Glial cell activation	4.92E-02	7.66	MAPT; TRPV1; IL33; TYROBP; SNCA	rs17763658; rs9905037; rs16924291; rs4806237; rs200955273	3.77; 3.62; 3.14; 2.74; 2.52

The most significant SNPs negatively associated with learning and memory, specifically in mTBI patients, rs1383914 and rs10788317, map to the synaptic signaling gene alpha 1-adrenergic receptor (*ADRA1A*) and the growth hormone inducible transmembrane protein (*GHITM*, also known as TMBIM5 and MICS1 at the protein level) in the mitochondrial organization pathway, respectively. *ADRA1A* has been linked to working memory dysfunction in TBI rat models^50^. The SNP specifically has been linked to the incidence and impact of fibromyalgia, a chronic pain disorder related to common pain-related symptoms post-mTBI and burdens on working memory capacity^51–55^. *GHITM* is responsible for mitochondrial formation and calcium ion regulation to maintain cell metabolism and survival. It does this through binding to and regulating the activity of cytochrome c along with Parkinson’s disease-associated intermembrane mitochondrial protein CHCHD2^56,57^. On the other hand, rs1250244 and rs1250231, which map to Fibronectin 1 (*FN1*), demonstrate a positive association with learning specifically in the mTBI group but no association in the OI group. FN1 is a major downstream effector of transcription factor 4 in the cortex and hippocampus to influence neuronal positioning in development and spatial learning and reasoning^58^. It has been shown to play an important role in mTBI recovery as well, thereby carrying promise as a potential TBI therapeutic target^59,60^. Plasma fibronectin demonstrated neuroprotective effects following TBI, with mice deficient in the protein performing significantly worse in motor and cognitive tasks^61^. These SNPs did not show association with NPC3 in the OI group. Altogether, our findings support that the top SNPs relating to synaptic signaling, neuronal projection, and mitochondrial function interact with mTBI, but not OI, to affect learning and memory.

### Cell type-specific network analysis reveals key regulators and networks governing neuronal repair pathways in the hippocampus and prefrontal cortex

To dissect the cell types and gene regulatory networks (GRNs) responsible for the significant pathways enriched for SNP-by-injury interaction in relevant brain regions, we constructed cell type-specific GRNs and performed Mergeomics’ Key Driver Analysis (KDA)^62^ on the significant mTBI-mediated genetic pathways. Given the extensive mTBI studies with mouse models^9–12^ that recapitulate many aspects of human pathology and the wide availability of high-quality reference cell type characterization in TBI-affected brain regions in the Allen Brain Atlas^63^, we inferred cell type-specific GRNs from scRNA-seq data from the mouse hippocampus and cortex. We inferred gene regulatory programs that define each cell type using SCING, a gradient-boosting and bootstrapping-based global GRN construction method^64^. We then performed KDA on the enriched GO-BP pathways using cell type GRNs from the hippocampus and various cortical regions (prelimbic, infralimbic, and orbital cortex; frontal pole and secondary motor cortex; and anterior cingulate cortex) (Table 2 and Supplementary Data 8). We focused on these brain regions due to their relevance to TBI. KDA employs a permutation approach on the GRN topology to identify hub genes, or “key drivers” (KD), that are highly connected to genes in the biological pathways of interest.

**Table 2:** Top key drivers for the significant mTBI-interaction pathways in hippocampal and cortical cell types

Key driver	Cell type	Pathway	KDA FDR	KDA enrichment	Top SNPs in SNP-by-mTBI interaction term	-log10 (GWAS P)
**Hippocampus**						
CPLX2	Excitatory	Regulation of trans synaptic signaling	9.06E-06	9.6	rs13174727; rs11134935; rs999065; rs11134936; rs13158114	1.06e-03; 1.27e-03; 1.45e-03; 6.12e-03; 6.88e-03
PJA2	Excitatory	Regulation of trans synaptic signaling	6.31E-05	8.03	rs9326769; rs1594076; rs2416213; rs7705657; rs2914711	2.38e-03; 4.42e-03; 4.45e-03; 7.03e-03; 7.47e-03
MAPT	Excitatory	Regulation of trans synaptic signaling	1.00E-02	5.63	rs17763658; rs9890016; rs73313152; rs9896752; rs62061733	1.71e-04; 1.57e-03; 4.05e-03; 8.59e-03; 2.69e-02
APP	Excitatory	Regulation of trans synaptic signaling	3.13E-02	5.63	rs440666; rs71317439; rs71317438; rs1580939; rs2211769	1.51e-02; 2.47e-02; 2.95e-02; 2.96e-02; 2.99e-02
YWHAG	Excitatory	Regulation of trans synaptic signaling	3.13E-02	6.5	rs2868371; rs2908195; rs2908203; rs2070804; rs62476798	1.32e-02; 5.99e-02; 8.38e-02; 9.58e-02; 1.37e-01
NDUFS6	Inhibitory	Mitochondrion organization	9.58E-08	9.72	rs6859291; rs904756; rs6555014; rs4296839; rs16884543	1.15e-02; 6.50e-02; 8.12e-02; 8.23e-02; 8.53e-02
COX5A	Inhibitory	Mitochondrion organization	3.12E-04	11.41	rs11638576; rs78324724; rs8033883; rs7175135; rs6495129	1.27e-01; 1.30e-01; 1.79e-01; 1.79e-01; 2.36e-01
NDUFC1	Inhibitory	Mitochondrion organization	4.45E-04	7.02	rs34711204; rs6814602; rs6536006; rs1901173; rs7682630	6.72e-03; 4.00e-02; 5.01e-02; 7.48e-02; 7.74e-02
COX17	Inhibitory	Mitochondrion organization	4.87E-04	8.3	rs149967762; rs71325395; rs13076819; rs4687877; rs2280580	1.10e-02; 1.62e-02; 1.72e-02; 1.96e-02; 2.91e-02
NDUFA7	Inhibitory	Mitochondrion organization	6.51E-04	8.33	rs448652; rs7248963; rs12982876; rs34661506; rs112110995	8.31e-02; 1.01e-01; 1.02e-01; 1.02e-01; 1.02e-01
PSAP	Inhibitory	Regulation of trans synaptic signaling	5.76E-03	12.13	rs9415055; rs9415050; rs118122154; rs7092990; rs115467618	3.43e-03; 4.52e-03; 1.69e-02; 2.23e-02; 2.44e-02
TSPAN2	Oligodendrocyte	Glial cell development	6.56E-03	36.74	rs6537851; rs74113933; rs12132759; rs6679900; rs3820630	3.95e-02; 8.14e-02; 9.05e-02; 9.99e-02; 1.05e-01
MOG	Oligodendrocyte	Glial cell development	1.57E-02	22.61	rs9258119; rs9257928; rs29273; rs29270; rs45617437	3.98e-02; 5.18e-02; 6.15e-02; 6.99e-02; 6.99e-02
CLDN11	Oligodendrocyte	Glial cell development	2.12E-02	36.74	rs56226070; rs2901623; rs4955726; rs13434085; rs34814917	2.83e-03; 1.43e-02; 2.53e-02; 2.84e-02; 3.49e-02
CNP	Oligodendrocyte	Glial cell development	2.12E-02	40.08	rs11079027; rs57825218; rs199922737; rs62076888; rs78071022	5.74e-02; 6.59e-02; 7.07e-02; 8.91e-02; 1.06e-01
**Anterior cingulate cortex**						
GRM7	Excitatory	Regulation of trans synaptic signaling	6.72E-03	10.38	rs712793; rs712774; rs34735406; rs712783; rs41361551	9.94e-04; 1.39e-03; 3.65e-03; 4.92e-03; 6.38e-03
NSF	Excitatory	Regulation of trans synaptic signaling	6.72E-03	10.92	rs199496; rs929254; rs199528; rs74939963; rs113839801	5.33e-03; 1.39e-02; 4.13e-02; 4.36e-02; 4.45e-02
GRM5	Excitatory	Regulation of trans synaptic signaling	1.22E-02	15.38	rs308804; rs1552739; rs56859701; rs973716; rs11826393	1.07e-04; 4.06e-04; 4.08e-04; 4.08e-04; 4.14e-04
SV2A	Inhibitory	Regulation of trans synaptic signaling	3.23E-05	7.52	rs6587723; rs6696191; rs577935; rs626785; rs147211319	5.88e-02; 9.06e-02; 1.02e-01; 1.43e-01; 1.61e-01
GRM5	Inhibitory	Regulation of trans synaptic signaling	6.53E-05	13.27	rs308804; rs1552739; rs56859701; rs973716; rs11826393	1.07e-04; 4.06e-04; 4.08e-04; 4.08e-04; 4.14e-04
NSF	Inhibitory	Regulation of trans synaptic signaling	3.62E-02	9.34	rs199496; rs929254; rs199528; rs74939963; rs113839801	5.33e-03; 1.39e-02; 4.13e-02; 4.36e-02; 4.45e-02
CHL1	Oligodendrocyte	Regulation of trans synaptic signaling	9.76E-03	15.89	rs4685713; rs11715727; rs9310805; rs13086886; rs77030744	4.75e-03; 1.39e-02; 2.11e-02; 2.29e-02; 2.54e-02
TSPAN2	Oligodendrocyte	Glial cell development	9.76E-03	43.97	rs6537851; rs74113933; rs12132759; rs6679900; rs3820630	3.95e-02; 8.14e-02; 9.05e-02; 9.99e-02; 1.05e-01
**Prelimbic infralimbic orbital cortex**						
CX3CL1	Excitatory	Regulation of trans synaptic signaling	2.58E-03	11.49	rs72784878; rs223880; rs148546737; rs4784801; rs757307	4.99e-02; 5.35e-02; 6.13e-02; 6.40e-02; 6.68e-02
GRIN2A	Excitatory	Regulation of trans synaptic signaling	8.37E-03	13.44	rs7189919; rs8060390; rs56064109; rs7201288; rs8055473	1.14e-02; 1.93e-02; 1.94e-02; 2.21e-02; 2.25e-02
MEF2C	Inhibitory	Regulation of trans synaptic signaling	2.52E-02	11.65	rs302482; rs302484; rs80043958; rs244752; rs244751	2.94e-02; 4.09e-02; 5.40e-02; 6.14e-02; 6.14e-02
GRIA4	Inhibitory	Regulation of trans synaptic signaling	5.29E-04	9.65	rs72979301; rs78442630; rs675091; rs17391463; rs10895891	1.24e-02; 2.15e-02; 3.37e-02; 5.37e-02; 5.59e-02
ATP5PD	Inhibitory	Mitochondrion organization	5.94E-03	7.2	None	None
MOG	Oligodendrocyte	Glial cell development	4.82E-02	32.78	rs9258119; rs9257928; rs29273; rs29270; rs45617437	3.98e-02; 5.18e-02; 6.15e-02; 6.99e-02; 6.99e-02
**Frontal pole-secondary motor cortex**						
ITPR1	Excitatory	Regulation of trans synaptic signaling	9.57E-03	7.42	rs6765702; rs73004812; rs2633717; rs73004818; rs73003098	1.91e-03; 2.86e-03; 3.19e-03; 3.37e-03; 3.51e-03
NSF	Excitatory	Regulation of trans synaptic signaling	9.93E-03	10.3	rs199496; rs929254; rs199528; rs74939963; rs113839801	5.33e-03; 1.39e-02; 4.13e-02; 4.36e-02; 4.45e-02
MOG	Oligodendrocyte	Glial cell development	1.64E-02	31.18	rs9258119; rs9257928; rs29273; rs29270; rs45617437	3.98e-02; 5.18e-02; 6.15e-02; 6.99e-02; 6.99e-02

**Fig. 3:**
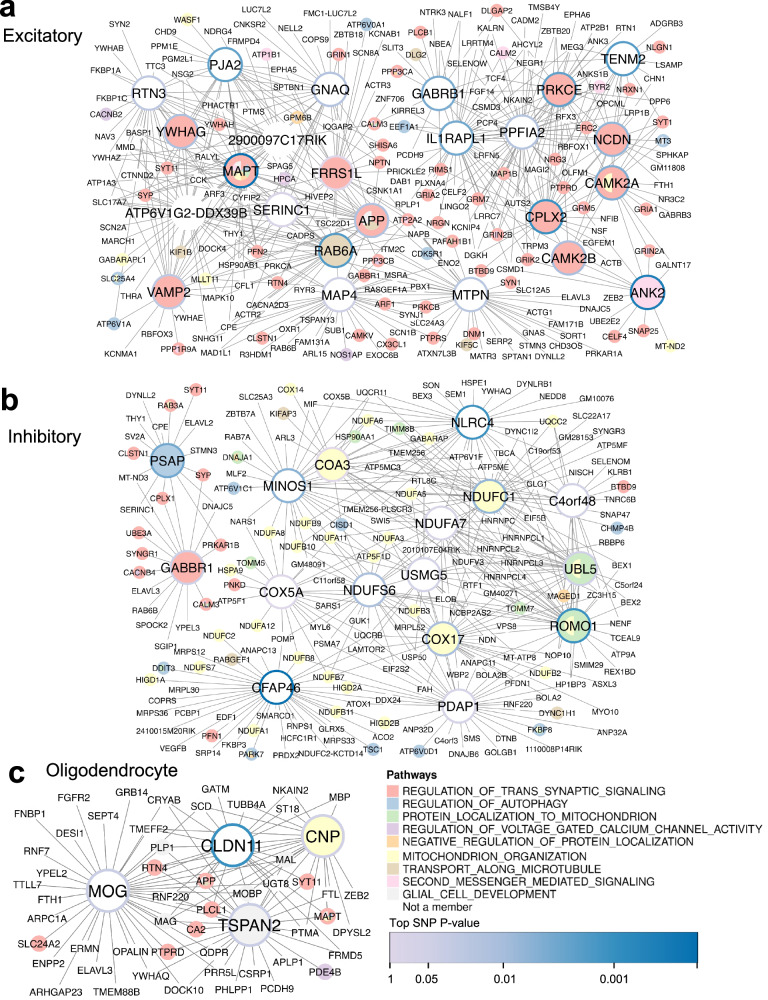
Hippocampus cell type gene regulatory networks of learning and memory pathways that interact with mTBI. Larger nodes (genes) represent key drivers (KDs) of pathways, and smaller nodes are the key driver neighbors. Node colors correspond to pathways. Node borders correspond to the GWAS *p* value of the top-mapped SNP in the SNP-by-mTBI interaction term. **a** Excitatory neuron KDs contain genes in the synaptic signaling pathway. **b** Inhibitory neuron KDs are involved mainly in mitochondrial organization. **c** Oligodendrocyte KDs are involved in myelination and glial cell development.

Using the hippocampus cell type GRNs, we identified numerous KDs at FDR <5% that regulated distinct processes in excitatory and inhibitory neurons, as well as in oligodendrocytes (Fig. 3). In the excitatory neuron GRN, KDs are involved in trans-synaptic signaling (e.g., *CPLX2;* FDR = 9.06e-6). Numerous hub genes in this network are known to be associated with Alzheimer’s disease (AD) pathology (e.g., *MAPT, APP, PJA2, YWHAG*) (Fig. 3a). *MAPT* (FDR = 0.01) encodes the tau protein responsible for neurofibrillary tangles and *APP* (FDR = 0.031) encodes the amyloid precursor protein that forms amyloid plaques in AD patients^65–67^. *PJA2* (FDR = 6.31e-5), is essential for long-term memory and synaptic plasticity, and inhibits *APP, MAPT*, and gamma-secretase activating protein *GSAP* expression in mice^68^. Also, *YWHAG* (FDR = 0.031) is a biomarker for AD^69^. The inhibitory neuron KDs mainly regulate the mitochondrial organization pathway with many KDs from the NADH dehydrogenase Complex 1 gene family, such as *NDUFS6* (FDR = 9.58e-8), and the cytochrome c oxidation family like *COX5A* (FDR = 3.12e-4) (Fig. 3b). Lastly, the oligodendrocyte network contains KDs promoting glial cell development, specifically through myelination genes *TSPAN2* (FDR = 6.56e-3), *MOG* (FDR = 0.016), *CLDN11* (FDR = 0.021), and *CNP* (FDR = 0.021) (Fig. 3c).

**Fig. 4:**
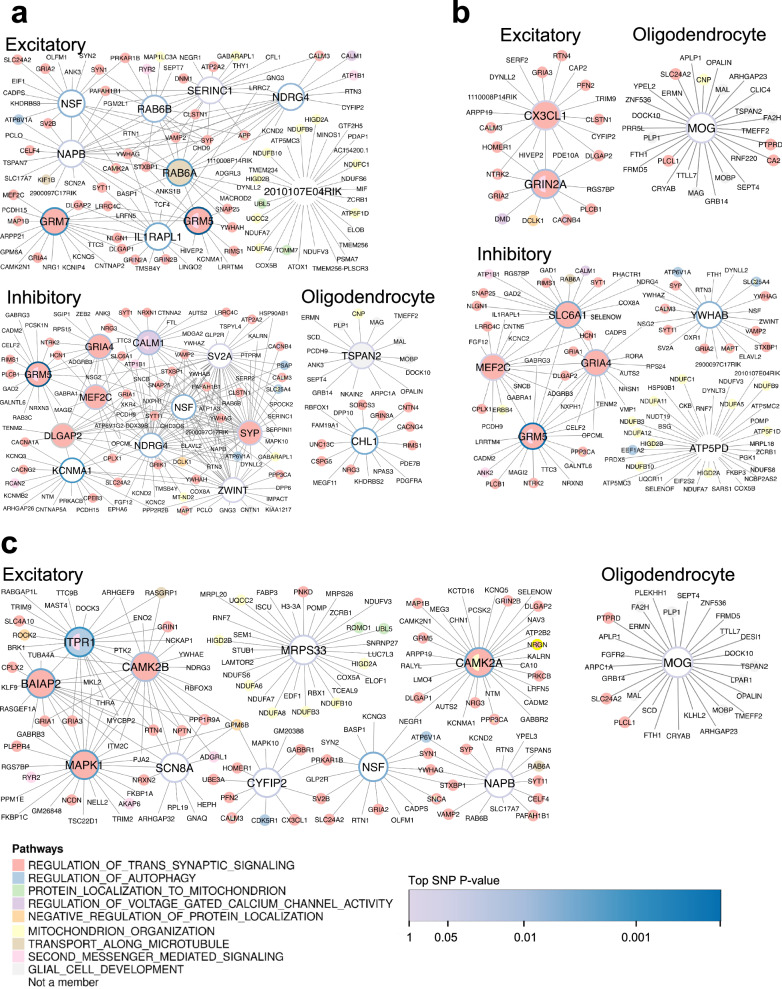
Cortex cell type key driver GRNs of learning and memory pathways. Larger nodes (genes) represent key drivers (KDs) of pathways, and smaller nodes are the key driver neighbors. Node colors correspond to pathways. Node borders correspond to the GWAS *p* value of the top-mapped SNP. **a** Anterior cingulate cortex networks. Excitatory and inhibitory neurons contain KDs for the synaptic signaling pathway, while the oligodendrocyte KDs additionally regulate glial cell development. **b** Prelimbic, infralimbic, orbital cortex networks. Both neuronal cell types drive synaptic signaling, and inhibitory neurons contain a KD for mitochondrial organization. Oligodendrocytes contain MOG as the single KD for glial cell development. **c** Frontal pole, secondary motor cortex networks. Only excitatory neurons contain KDs for both synaptic signaling and mitochondrial organization, and MOG remains a KD for glial cell development.

Among the cortical regions, the anterior cingulate cortex contains the most KDs. For the excitatory neurons, *GRM7* (FDR = 6.72e-3) and *NSF* (FDR = 6.72e-3) are the top KDs for the trans-synaptic signaling pathway, whereas in inhibitory neurons *SV2A* (FDR = 3.23e-5) is the top KD for the same pathway (Fig. 4a). For oligodendrocytes, *CHL1* (FDR = 9.76e-3) and *TSPAN2* (FDR = 9.76e-3) are the KDs for trans-synaptic signaling and glial cell development, respectively (Fig. 4a). Within the prelimbic, infralimbic, and orbital cortex networks, a majority of KDs are significant for the trans-synaptic signaling pathway (Fig. 4b). These include the excitatory neuron KDs *CX3CL1* (FDR = 2.58e-3) and *GRIN2A* (FDR = 8.37e-3), and the inhibitory neuron KDs *GRIA4* (FDR = 5.29e-04) and *ATP5PD* (FDR = 5.94e-03). *MOG* (FDR = 0.048) is the only oligodendrocyte KD for the glial cell development pathway in the prelimbic, infralimbic, and orbital cortex networks. The frontal pole and secondary motor cortex GRNs (Fig. 4c) also yielded KDs for the trans-synaptic signaling pathway, including excitatory neuron KDs *ITPR1* (FDR = 9.57e-03) and *NSF* (FDR = 9.93e-3). *MOG* (FDR = 0.016) remains the oligodendrocyte KD for glial cell development in the frontal pole and secondary motor cortex GRNs (Fig. 4c).

Across brain regions, *MOG* was the most significant KD for the glial cell development pathway for the hippocampus, prelimbic-infralimbic-orbital cortex, and frontal pole-secondary motor cortex, and was the second most significant KD for the pathway in the anterior cingulate cortex. *MOG* produces a membrane protein on oligodendrocytes, and using a human leukocyte antigen linked to a MOG mouse peptide was found to improve neural deficits in a mouse model of TBI^70^.

Our brain region- and cell type-specific network analysis revealed potential cell types and gene regulators of the significant biological pathways enriched for SNP-by-mTBI interaction GWAS signals for neurocognition, highlighting the role of excitatory and inhibitory neurons and oligodendrocytes in mTBI and their respective gene regulators of metabolic, synaptic, and glial pathways critical for neural repair and function.

### A polygenic risk score (PRS) of top pathway SNPs demonstrates clinical relevance and significant modeling improvement

To evaluate the clinical relevance and potential utility of the interaction SNPs and pathways from our genetic analysis, we created pathway-based PRS scores for each patient based on the weighted sum of effect sizes and risk allele counts of the SNPs from the top biological pathways (Supplementary Data 9) and fit linear models to predict NPC3 scores from the PRS. We generated an additive PRS based on the additive GWAS summary statistics and an interaction PRS based on the summary statistics from the interaction GWAS for NPC3 to compare the performance of the linear models with or without an interaction term (details in Methods).

**Fig. 5:**
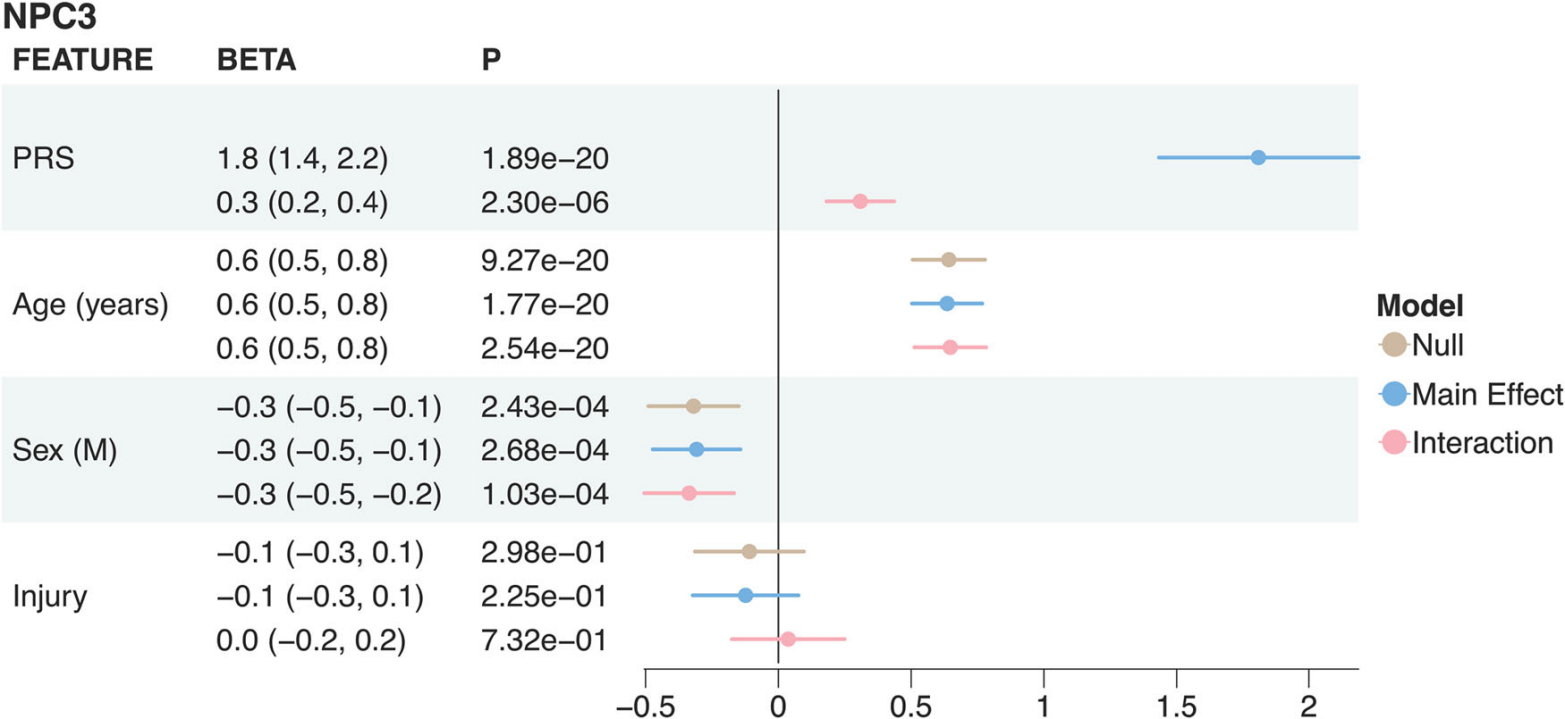
Pathway-based PRS for NPC3 in ABCD. Forest plot of pathway-based polygenic risk score and covariates across NPC3 predictive models. Colors are assigned to the three linear models (null model without PRS in beige, additive model with additive PRS in blue, interaction model with interaction PRS in red). The x-axis of the plot delineates the effect size of the linear models, accompanied by a 95% confidence interval, enabling direct comparison of the linear models for each feature.

The interaction model contained the highest predictive accuracy for NPC3 with an *R*^2^ of 0.215, followed by the additive (*R*^2^ = 0.208) and the null model without PRS (*R*^2^ = 0.1584) (Fig. 5). Compared to the null model, both the additive and interaction models significantly improved the prediction accuracy (likelihood ratio test *p* < 0.001 for both). Between the two PRS models (non-nested), using the interaction PRS improves model performance based on Akaike Information Criterion (AIC = 5316) compared to the additive PRS (AIC = 5329). This is a significant improvement according to Burnham and Anderson, 2003, who found an AIC difference of at least ten points between non-nested models to be significant^71^. To address the potential of overfitting, we performed a fivefold cross-validation and observed the same order of model performance across the three model types (interaction > additive > null), and no significant difference in accuracy between training and validation sets (Supplementary Data 10). Furthermore, accounting for the SNP-by-mTBI interaction with the interaction PRS reveals a greater effect size on NPC3 score than when only considering the main genetic effect in the additive PRS. Both PRS contain a greater effect on learning and memory performance than sex and a slightly weaker effect than age. The injury variable was not significant in any model, consistent with a recent report that mTBI is not associated with cognitive performance in ABCD when compared to OI controls^72^. The significant improvement of the interaction model suggests that SNPs in the neuronal repair, synaptic signaling, mitochondrial function, and glial cell activation pathways interact with mTBI and are predictive of mTBI recovery in learning and memory.

## Discussion

mTBI remains a leading form of brain injury, yet its effects on child neurocognitive development remain unclear, particularly given the high variability in mTBI outcomes^1–7^. With the ABCD cohort, we performed the largest and most granular multiomic systems analysis of mTBI outcome to date, using an innovative systems biology pipeline that integrates interaction GWAS signals with cell type-resolved regulatory networks to identify modifiers of learning and memory performance affected by mTBI at the network level. Our gene-by-mTBI interaction GWAS on NPC3, a neurocognition measure, detected SNPs with differential effects on neurocognition based on whether the patient sustained an mTBI or OI. Performing threshold-free GWAS integration with pathways and cell type-specific cortical and hippocampal networks based on an omnigenic disease model uncovered key cell type and gene drivers orchestrating neuronal and glial development and mitochondrial pathways. Furthermore, condensing these pathways into a polygenic risk score provides further insight into the clinical utility for predicting neurocognitive outcomes for individuals with mTBI and orthopedic injury. This study highlights the importance of leveraging unbiased network approaches to elucidate the underlying brain regions, cell types, biological mechanisms and key gene regulators of mTBI recovery separate from general injury recovery.

Compared to previous studies on mTBI investigating genetic effects on neurocognitive and behavioral domains, the current study is a departure from the practice of using prior genetic knowledge to restrict the search for candidate SNPs and biological pathways^17,24,29^. Here, we demonstrate the advantage and power of biological discovery with data-driven network modeling approaches that surveyed all SNPs and gene ontologies and networks to explore mTBI- and genetically mediated neurocognitive effects and the interactions between the two. First, we identified novel genetic mediators of many known biological pathways in mTBI pathology and brain function, such as neuronal repair, synaptic signaling, mitochondrial organization, and glial cell development^73,74^. For example, we identified the top SNP for the trans-synaptic signaling pathway gene *ADRA1A* (rs138914), as a potential genetic regulator of mTBI effect on learning and memory, though previously studied in fibromyalgia^51–55^. We also report the top SNP rs10788317 that maps to *GHITM* in the mitochondrial organization pathway, which is previously associated with a known Parkinson’s disease gene, *CHCHD2*^56,57^. While these genes and variants have been studied in the context of these neurological disorders, we propose them for further investigation in their role in influencing learning and memory in mTBI. Furthermore, our human genetic analysis also provides human validation of genes previously associated with poor TBI prognosis in rodent models, such as *ADRA1A* and *FN1*, which were involved in trans-synaptic signaling and cell adhesion and migration pathways in our findings. Occurring in 74% of the 137 significant biological pathways, *MAPT*, which encodes microtubule-associated protein Tau, is central to neuronal growth and plasticity to influence learning, and serves as a known biomarker for TBI^67,75,76^. Emerging studies on the function of mitochondria in memory formation coincide with the high prevalence of mitochondrial pathways in our GWAS^77–81^.

In addition to employing a pathway-based analysis, the incorporation of GRN inference with scRNA-seq data of cortical and hippocampal regions from the Allen Brain Atlas elucidated the integral role of these genetic mechanisms to coordinate unique cell type regulatory pathways, drive different aspects of mTBI pathology, and control the disease’s impact on learning and memory performance. We discovered key differences between inhibitory and excitatory neuron activity in post-injury neurocognitive outcomes. In the hippocampus, the inhibitory neuron GRN for SNP-by-mTBI interaction pathways mainly contains KDs involved in mitochondrial function, such as *NDUFS6* and *COX5A*, which have not been studied in the context of TBI. Mitochondrial dysfunction has been associated with GABA-ergic-glutamatergic neuron imbalance, which may initiate neuropsychiatric disorders^77,78,82–84^. The mitochondrial complexes 1 and 4, which include *NDUFS6* and *COX5A*, respectively, are also genetically associated with Alzheimer’s disease and memory impairment^79–81^. While mitochondrial dysfunction is a known mechanism of TBI pathology, our network analysis of the SNP-mTBI interaction pathways emphasizes the potential role of mitochondrial dysregulation at complexes 1 and 4 in inhibitory GABA-ergic neurons to affect learning and memory. On the other hand, the excitatory neuron gene regulatory network focuses on synaptic signaling with key drivers like *APP, MAPT*, and *PJA2*, which are known Alzheimer’s disease risk genes. These results not only align with previous studies of brain injury increasing risk for neurodegenerative disease^65–68,85^ but also propose potential shared genetic mechanisms that regulate neuronal activity in both pathologies, where genetic variants in these genes confer cognitive vulnerability, and mTBI may further interact with this vulnerability. *APP*, for instance, is cleaved to release sAPPα fragments to promote acute neuronal repair in mTBI animal models while also depositing amyloid-β plaques, which is one of the main pathologies of Alzheimer’s disease^66,86,87^. Likewise, MAPT’s dysregulation in mTBI provides the possibility of neurofibrillary tangles^67,75,76^. The oligodendrocyte network identifies glial development genes as key drivers. *MOG*, the top key driver in both hippocampus and cortical regions, has been studied for neuroprotective effects in modulating pathogenic immune functions in brain injury and stroke when administered as a partial MHC class II construct^70,88–90^. Our network analysis adds further evidence supporting its functional role in modifying mTBI-induced cognitive outcomes. Furthermore, while previous studies focus on general gene networks without tissue and cellular context, this study emphasizes the importance of considering the brain region and cell type contexts in understanding the complexity of TBI pathology. It is important to note that these key drivers were predicted statistically based on cell type network centrality. While they may serve as novel targets, they must be tested experimentally to confirm causal roles in affecting learning and memory post-mTBI.

Lastly, we tested the clinical significance and utility of polygenic risk scores of the top gene-by-mTBI interaction pathways in forecasting learning performance. Including PRS based on the additive or interaction GWAS into clinical predictive models demonstrated increased performance accuracy over the null model without the PRS. The performance increase with the interaction PRS suggests potential clinical utility of this gene-by-injury component in predicting learning and memory. Other approaches can be also incorporated to optimize the PRS, such as shrinkage, to better assess its clinical utility^91–93^. Nonetheless, the performance improvement in the interaction model is modest, and our cross-validation results also demonstrate greater variability in accuracy in validation sets, which indicates overfitting to the training sets. For these reasons, there is uncertainty in their translatability to a clinical setting. Therefore, it is important to externally test the generalizability of our PRS models to independent pediatric cohorts when available.

We also acknowledge that our study focuses on the genetic mechanisms that interact with brain injury to affect neurocognitive outcome compared to orthopedic injuries. As such, our findings should be interpreted within the context of injured participants, and it would be of interest to pursue comparisons with non-injured children in ABCD in future studies.

Overall, our study represents one of the first data-driven, systems-level explorations of gene-by-injury effects in pediatric mTBI. This systems biology framework is established through the comprehensive integration of gene-by-mTBI GWAS interaction effects in the ABCD cohort, existing genetic studies of a broad range of neurological traits related to mTBI and cognition, biological pathways, and brain cell type-specific networks to reveal molecular mechanisms governing cell type processes involved in mTBI pathology regarding learning and memory. The identification of cell type-specific key driver genes and pathways provides potential targets for testing their causal and functional effects in TBI animal models and warrants future investigations into their utility and clinical translation.

## Methods

### Study design

To test the omnigenic model on mTBI-interacting SNPs, we first performed a gene-by-mTBI interaction GWAS on neurocognition in the ABCD baseline cohort to identify SNPs with a differential effect on neurocognitive outcome between children with OI and those with mTBI in ABCD. The selection of OI as the reference group is common in mTBI studies to control for factors that predispose children to accidents, as well as the psychological trauma associated with an injury and its treatments^48^. As our hypothesis emphasizes the role of the full range of SNPs with strong, moderate, or weak effects in influencing mTBI outcome through core and peripheral gene networks, we used the whole spectrum of GWAS summary statistics. We aimed to identify the biological pathways and networks mediating the gene-by-mTBI interactions by comparing the distribution of gene-by-mTBI interaction association strengths of SNPs mapped to biological pathways compared to random SNP sets, a method implemented in Mergeomics’ MSEA^62^. We further leveraged neuronal and glial gene regulatory networks in cortical and hippocampal regions to identify the cell type-specific network mechanisms that drive these pathways, providing greater resolution into cell type-specific mechanisms in mTBI pathophysiology. Beyond mechanistic interpretation, we also leveraged the significant biological pathways and their associated SNPs to calculate pathway-based polygenic risk scores as an interpretable measure of genetic risk prediction for neurocognitive outcome in the presence or absence of mTBI.

### Clinical data acquisition

Patient data were retrieved from the ABCD version 5.1 release. The ABCD study is an ongoing study including 11,878 children ages 9–10 across 21 research sites in the United States. Children undergo annual assessments for neurocognitive, behavioral, psychological, and biological endpoints. All ABCD data were accessed through the National Institute of Mental Health (NIMH) data archive (NDA).

Demographics: We used the age, sex, and ethnicity variables in our analysis. Race/ethnicity was reported as a categorical variable with levels White, Black, Hispanic, Asian, and Other/Mixed.

Injury grouping: Injury groups were defined based on two ABCD measures: the Ohio State Traumatic Brain Injury Screening for mTBI and the Medical History Questionnaire for OI^94,95^. We considered mTBI as patients sustaining a head injury with memory loss and/or loss of consciousness for under 30 minutes. The orthopedic injury (OI) group was assigned by parent reports on whether the child had ever visited a medical office for a bone fracture.

Neurocognitive outcome: We used neurocognitive performance scores from the NIH Toolbox Cognitive Battery (https://nihtoolbox.org/), the Rey Auditory Verbal Learning Test, and the Little Man Task. To obtain learning and memory scores in the participants, we followed the Bayesian probabilistic principal component analysis conducted in Thompson et al^32^. This method aggregates nine administered test scores (NIH Toolbox measures Picture Vocabulary Task, Oral Reading Recognition Task, Pattern Comparison Processing Speed Test, List Sorting Working Memory Test, Picture Sequence Memory Test, Flanker Task, and Dimensional Change Card Sort Task; Rey Auditory Verbal Learning Task; and Little Man Task) into three neurocognitive principal components (NPCs) that correlate with general cognition (NPC1), executive function (NPC2), and learning and memory (NPC3). The learning and memory NPC3 contained the highest principal component loadings for the Toolbox Picture Sequence Memory Task, Toolbox List Sorting Working Memory Task, and Rey Auditory Verbal Learning Task, and was the primary focus of the current study. We chose to use the principal components instead of individual phenotypic traits to capture the overall learning and memory performance and to reduce the multiple testing burden.

Genetic data: We used the imputed genetic data and genetic principal components (PCs) from ABCD. Data collection, quality control, linkage disequilibrium, genetic relatedness filtering, and PC calculation on patient genotype data are documented in Fan et al.^96^, as follows: ABCD collected ~500,000 genetic variants for 11,666 patients using the Affymetrix Smokescreen array and imputed remaining SNPs using the TOPMED imputation panel under the GRCh38 genome build. The consortium also calculated genetic PCs using PC-AIR from the GENESIS R package to account for the multi-ancestry cohort. SNPs were pruned using GENESIS’ snpgdsLDPruning function with a 10e6 base pair sliding window and LD correlation threshold of 0.1, leaving 158,103 SNPs for PC calculation. The snpgdsIBDKING function was used to estimate kinship and identify 8177 unrelated patients to be used in PC-AIR. The genetic PCs of the remaining individuals were determined from the PC loadings.

Baseline cohort description: Of the 11,878 children enrolled in the ABCD project at baseline, 10,870 had available genotype and neurocognitive data. Of these, 8978 had no injury at baseline, 1469 reported an OI, and 423 were classified as having an mTBI according to the Ohio State Traumatic Brain Injury Assessment^94,95^. After excluding genetically related individuals, 1522 children (1180 OI and 342 mTBI) were included in our study. We used the NPC3 for our analysis.

### Genome-wide association study

GWAS was performed using PLINK version 1.9^97^. We used a minor allele frequency threshold of 0.05 to filter out rare SNPs. Covariates included in the GWAS model were patient age, sex, injury type, and the top 20 genetic principal components. Since the NPC3 score in ABCD is site-adjusted^32^, we did not include site as a covariate in our GWAS. Among the mTBI (*n* = 423) and OI (*n* = 1469) children, we used the kinship matrix calculated by Fan et al.^96^ to subset the cohort to those with genetic relatedness below 0.25, thus keeping one sibling at random, except in the case of a mTBI-OI sibing pair for which the mTBI sibling was kept. This resulted in 1522 children (1180 OI and 342 mTBI) who were included in our study. We ran GWAS on all unrelated mTBI and OI patients at baseline under two model formulations: the additive and interaction models. The additive model measures the main SNP effect, and the interaction model includes the main and injury-by-SNP interaction effect.

### Validation of the main effect GWAS in ABCD using independent working memory GWAS with large sample sizes

Though the ABCD baseline cohort of OI and TBI only contains 1522 unrelated patients, we validate its use for GWAS functional enrichment studies by correlating SNP effect size estimates (regression coefficients) with findings from preexisting GWAS studies on similar phenotypes. Since the NIH toolbox measures for learning and memory relate to working memory. We identified five GWAS studies^34–38^ on verbal working memory, short-term memory, and spatial working memory and correlated their summary statistics with our ABCD GWAS. We performed Pearson correlation on the effect sizes of the overlapping SNPs between ABCD (NPC3) and each of the five large GWAS studies.

### Selection of TBI-related and unrelated SNP sets from the GWAS Catalog

To assess the validity and relevance of the GWAS, including mTBI-by-SNP interactions, we hypothesize that the genetic signals modifying the mTBI effect in learning and memory should be relevant to cognitive traits as well. Therefore, we collaborated with two neuroscientists independently to establish two sets of positive control traits (50 in each set; should be relevant to mTBI outcomes and neurocognition related traits such as cognitive ability and household income) and two sets of negative control sets (50 in each set; should be less relevant to mTBI and neurocognition, such as aortic diameter or liver enzyme levels) (Supplementary Data 5). These traits were selected from 2891 traits collected in the GWAS catalog^98^. Each of the neuroscientists relied on their accumulated individual career experiences in the field of neuroscience and knowledge of the neurological underpinnings of learning and memory performance in mTBI. Given the large number of traits screened and the separation of analyses between the neuroscientists, we did not overlap the selected traits or resolve any differences to keep them as independent control sets.

### SNP set enrichment analysis

We used the Mergeomics method to obtain SNP set enrichment statistics using full GWAS summary statistics^62^. LD clumping was performed using Mergeomics’ marker dependency filtering, excluding redundant SNPs with LD ≥ 0.5. SNPs were mapped to genes in pathways from Gene Ontology Biological Process from MSigDB and the GWAS Catalog^98,99^ using the hg38 reference genome based on a 50-kb distance threshold. We calculated the enrichment of SNPs passing multiple GWAS thresholds for each biological pathway using Mergeomics’ marker set enrichment analysis (MSEA), which uses a chi-like statistic and performs a permutation test on the SNP-pathway mappings to generate true and null enrichment statistics. We used Benjamini-Hochberg correction to control the false discovery rate at 0.05 to identify significant biological pathways for the SNPs.

### European-only gene-by-mTBI interaction GWAS on neurocognition and MSEA validation

To address potential residual population stratification in our interaction GWAS, we subsetted our main cohort into European participants, which is the largest ancestry group in ABCD, with 790 OI and 249 mTBI children. We conducted the gene-by-injury interaction GWAS on neurocognitive outcome using the same covariates as the main interaction GWAS across populations, as well as the same MSEA analysis to identify the biological pathways that a enriched for the European-only GWAS. Significant pathways were compared between the multi-ancestry analysis and the European-only analysis.

### Construction of cell type-specific GRNs using scRNA-seq data from the Allen Brain Atlas

We collected mouse single-cell RNA sequencing data from Allen Brain Atlas and classified brain regions and cell types based on the Allen Brain Institute’s published cell taxonomies^63^. We used Single Cell INtegrative Gene regulatory network inference (SCING) to construct global GRNs for each cell type^64^. From scRNA-seq data, SCING first mitigates cell sparsity by aggregating the expression of similar cells using Leiden clustering on the K-nearest neighbor graph for each cell type. To infer regulatory relationships, SCING trains gradient-boosting machines to predict target gene expression based on their co-expressed genes and uses feature importance measures of the feature genes to weigh directed network edges. For robustness, SCING bootstraps 100 subsets from the pseudo-bulk data, performs the GRN inference step for each subset to yield 100 intermediate GRNs, and aggregates recurring network edges among the intermediate GRNs to produce a final consensus GRN. We performed SCING for each of the cortical and hippocampal cell types from the 10X Chromium scRNA-seq data in the Allen Brain Atlas. Then we converted mouse gene names to human gene names using the Ensembl gene conversion table from the BiomaRt R package^100^.

### Key driver analysis (KDA) of biological pathways

Genetic mechanisms underlying TBI consequences of learning and memory were identified using KDA on each of the cortical and hippocampal cell type GRNs^62^. KDA uses the GRN topology to identify hub genes, or key drivers, with high connectivity to gene sets of interest, in this case, enriched biological pathways from MSEA analysis of the SNP-by-mTBI interaction GWAS. It identifies hub gene neighborhoods enriched for the gene sets through a chi-like statistic, and measures the significance of the enrichment based on the null distribution of enrichment scores generated from permuted networks. The significant hub genes at FDR < 5% are proposed as the key drivers of the mTBI-interacting pathways. We performed KDA on normal mouse hippocampus and prefrontal cortex cell type GRNs.

### Polygenic risk score modeling

We calculated pathway-based PRS from the MSEA results on the GWAS interaction model. The top 20 SNPs for each significant pathway in MSEA were selected as determined by GWAS *p* value for the SNP-by-mTBI interaction term. This process resulted in a collection of 345 unique SNPs, of which 41 were significantly associated with TBI but not OI patients when fitting injury-specific linear models on NPC3 (FDR ≤0.05). We used these SNPs to create our pathway-specific PRS (Supplementary Data 9). We repeated the same analysis for the additive GWAS to retrieve the SNPs for the additive PRS (Supplementary Data 9).

We used PLINK version 1.9 to calculate the PRS^97^. We calculated PRS with the additive GWAS effect sizes to obtain the additive PRS and the interaction GWAS effect sizes to obtain the interaction PRS. We calculated PRS scores for the same patients used in the GWAS. For predictive models, we built linear regression models on the NPC3 learning and memory variable. We included covariates of age, sex, and ethnicity in the model and compared the model performance of the following three models:

$${Null}\,{model}:\,{Phenotype}\, \sim \,{Injury}\,{type}+{Covariates}$$

$${Additive}\,{model}:\,{Phenotype}\, \sim \,{PR}{S}_{{add}}+{Injury}\,{type}+{Covariates}$$

$${Interaction}\,{model}:\,{Phenotype}\, \sim \,{PR}{S}_{int}+{Injury}\,{type}+{Covariates}$$

Injury type is an indicator of mTBI, with OI as reference. Covariates include self-reported race/ethnicity, age in months, and sex (M vs F). The PRS_add_ and PRS_int_ are derived from their respective GWAS models:

Additive GWAS model:

$$NPC3\, \sim \,{\beta }_{a}^{add}{X}_{a}+\,\mathop{\sum }\limits_{k=1}^{20}{\gamma }_{k}P{C}_{k}+{\beta }_{age}^{add}{Age}+{\beta }_{sex}^{add}Sex+{\beta }_{IG}^{add}IG+e$$

$${{\mathrm{PRS}}}_{{\mathrm{add}}}=\mathop{\sum }\limits_{a=1}^{A}{\beta }_{a}^{add}{X}_{a}$$

Interaction GWAS model:

$$NPC3\, \sim \,{\beta }_{i}^{int}{X}_{i}+{\beta }_{{gi}}^{add}{IG}{X}_{i}+\mathop{\sum }\limits_{k=1}^{20}{\gamma }_{k}P{C}_{k}+{\beta }_{age}^{int}{Age}+{\beta }_{sex}^{int}{Sex}+{\beta }_{IG}^{int}IG+e$$

$${{\mathrm{PRS}}}_{{\mathrm{int}}}=\mathop{\sum }\limits_{a=1}^{A\backslash I}{\beta }_{a}^{add}{X}_{a}+\mathop{\sum }\limits_{i=1}^{I}({\beta }_{i}^{int}{X}_{i}+{IG}{\beta }_{{gi}}^{int}{X}_{i})$$

where *A* represents the selected pathway SNPs from the additive GWAS, *I* represents the selected SNPs from the interaction GWAS, and *A\I* represents the SNPs in *A* but not in *I*.

Comparison of performance between models was performed using the likelihood ratio test for nested models (null vs additive, null vs interaction) and Akaike Information Criteria for non-nested models (additive vs interaction). To assess overfitting, we performed a stratified fivefold cross-validation by injury group with an 80/20 train-validation split for the null, additive, and interaction models. We compared the *R*^2^ distributions between training and validation folds using a two-sided paired *t*-test.

### Visualization

All plots were generated in R version 4.1.0, and all networks were generated with Cytoscape^101^. We visualized GRNs the direct neighbors of the significant key drivers in each cell type. Figure 1 was created using BioRender at https://biorender.com/orw6tm5 and licensed under CC BY 4.0.

### Ethics declaration and consent to participate

The ABCD study was approved by the institutional review board (IRB) of the University of California, San Diego (IRB# 160091), as well as the IRBs of each of the 21 data collection sites, in accordance with the Code of Federal Regulations on the Protection of Human Subjects (45 CFR 46). Since study participants were recruited at 9–10 years of age, informed consent was obtained from all parents and caregivers, and informed assent was obtained from participants. Data can be accessed through registration with the ABCD study at https://nda.nih.gov/abcd.

## Supplementary information

Supplementary Information

Supplementary Data 1

Supplementary Data 2

Supplementary Data 3

Supplementary Data 4

Supplementary Data 5

Supplementary Data 6

Supplementary Data 7

Supplementary Data 8

Supplementary Data 9

Supplementary Data 10

## Data Availability

All data pertaining to ABCD is available through the National Institute of Mental Health Data Archive (NDA). The Gene Ontology biological pathways were downloaded from Molecular Signature Databases (https://www.gsea-msigdb.org/gsea/msigdb/human/collections.jsp#C5). The single-cell RNA-seq data were downloaded from Allen Brain Atlas (https://portal.brain-map.org/atlases-and-data/rnaseq). Data analysis scripts can be found in our GitHub repository https://github.com/XiaYangLabOrg/ABCD_GWAS or by contacting the lead author.
