# Supplementary material for: Neuronal and glial networks interact with traumatic brain injury to modulate cognition in ABCD study
Source: NPJ Syst Biol Appl. 2026 Mar 13;12:60. doi: 10.1038/s41540-026-00681-8 (PMC13128933; doi:10.1038/s41540-026-00681-8)
Supplement: Supplementary file 1 — Supplementary Information [file 41540_2026_681_MOESM1_ESM.pdf]

Supplementary Information

Supplementary Data 1: NPC3 Additive GWAS Summary Statistics (P < 1e-5)

Supplementary Data 2: NPC3 Additive GWAS Effect Size Correlations with Positive and Negative Control Traits

Supplementary Data 3: NPC3 Interaction GWAS Summary Statistics (P < 1e-5)

Supplementary Data 4: Marker Set Enrichment (MSEA) Results on the GWAS Catalog for NPC3 GWAS Interaction Term

Supplementary Data 5: MSEA of TBI-associated GWAS Traits and Unrelated Negative Control GWAS Traits selected by 2 Neuroscientists

Supplementary Data 6: MSEA of Gene Ontology Biological Pathways for NPC3 GWAS Interaction Term

Supplementary Data 7: European-only MSEA of Gene Ontology Biological Pathways for NPC3 GWAS Interaction Term

Supplementary Data 8: Key Driver Analysis Results for Hippocampus and Cortex Cell Type Networks

Supplementary Data 9: SNPs Selected for Pathway-based PRS

Supplementary Data 10: Cross Validation Accuracies for Null, Additive, and Interaction Clinical Models

Supplementary Figures

Supplementary Figure 1: Additive GWAS summary statistics and validation with previous memory GWAS.

Supplementary Figure 2: Interaction GWAS summary statistics and validation with previous GWAS.

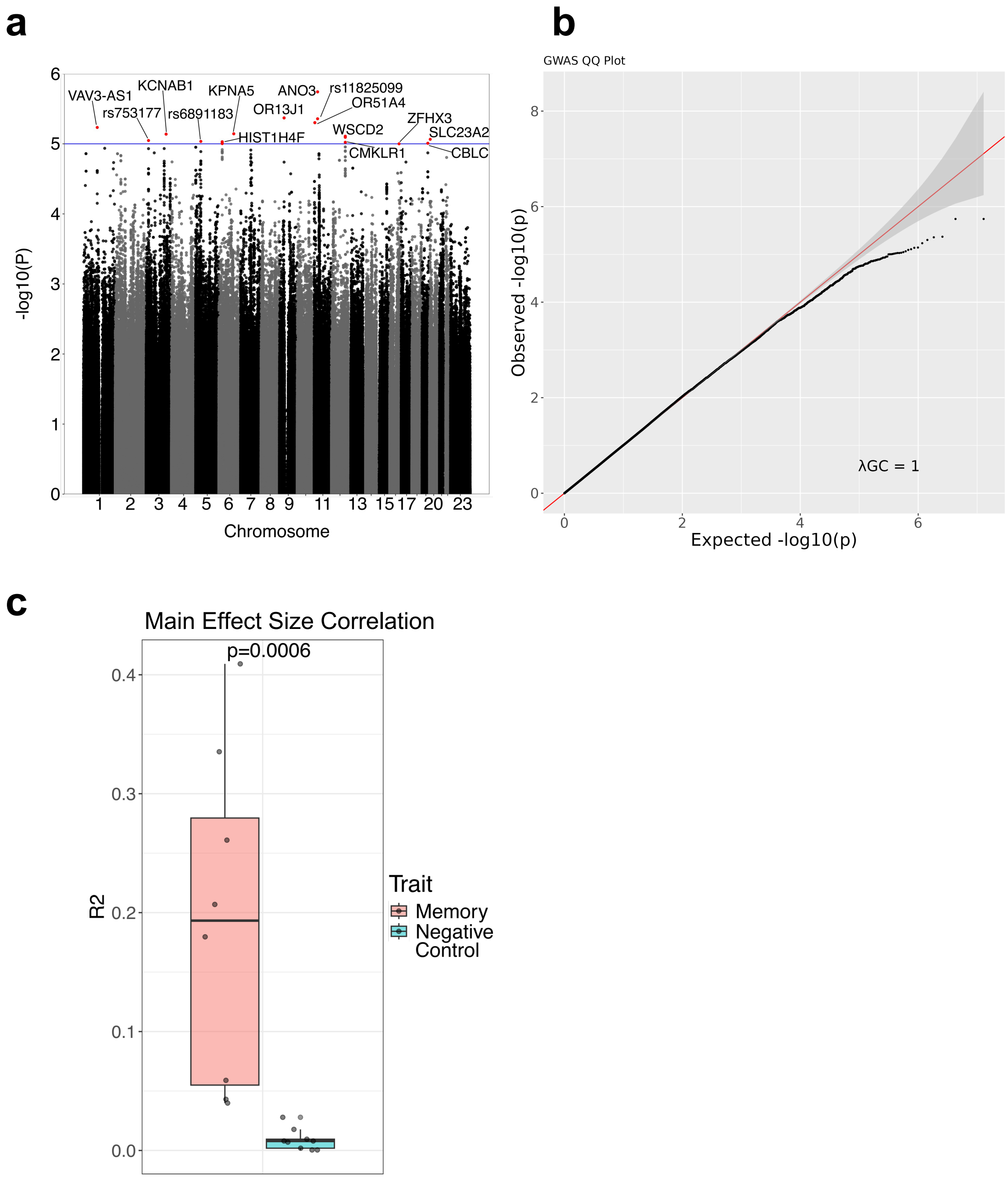

Supplementary Figure 1: Additive GWAS summary statistics and validation with previous memory GWAS. **a)** Manhattan Plot of GWAS additive model for the neurocognitive measure NPC3. Blue line is the genome wide suggestive threshold p=1e-5. **b)** QQ-plot of GWAS p-values. Genomic inflation factor of 1 indicates no systematic confounders, and the deflation for lower p-values (higher –log10(p)) from the expected is common for low sample sizes and over correction of genetic principal components. **c)** Pearson strength of correlation boxplots between ABCD additive GWAS and previous GWAS traits. Red boxplot consists of working, verbal, and spatial memory GWAS effect sizes, which is expected to have higher effect size correlation. Correlations were calculated for the overlapping SNPs between the ABCD additive GWAS and each Memory/Negative Control GWAS separately. Blue boxplot consists of phenotypes unrelated to memory or learning, where SNP effect sizes are expected to be less correlated.

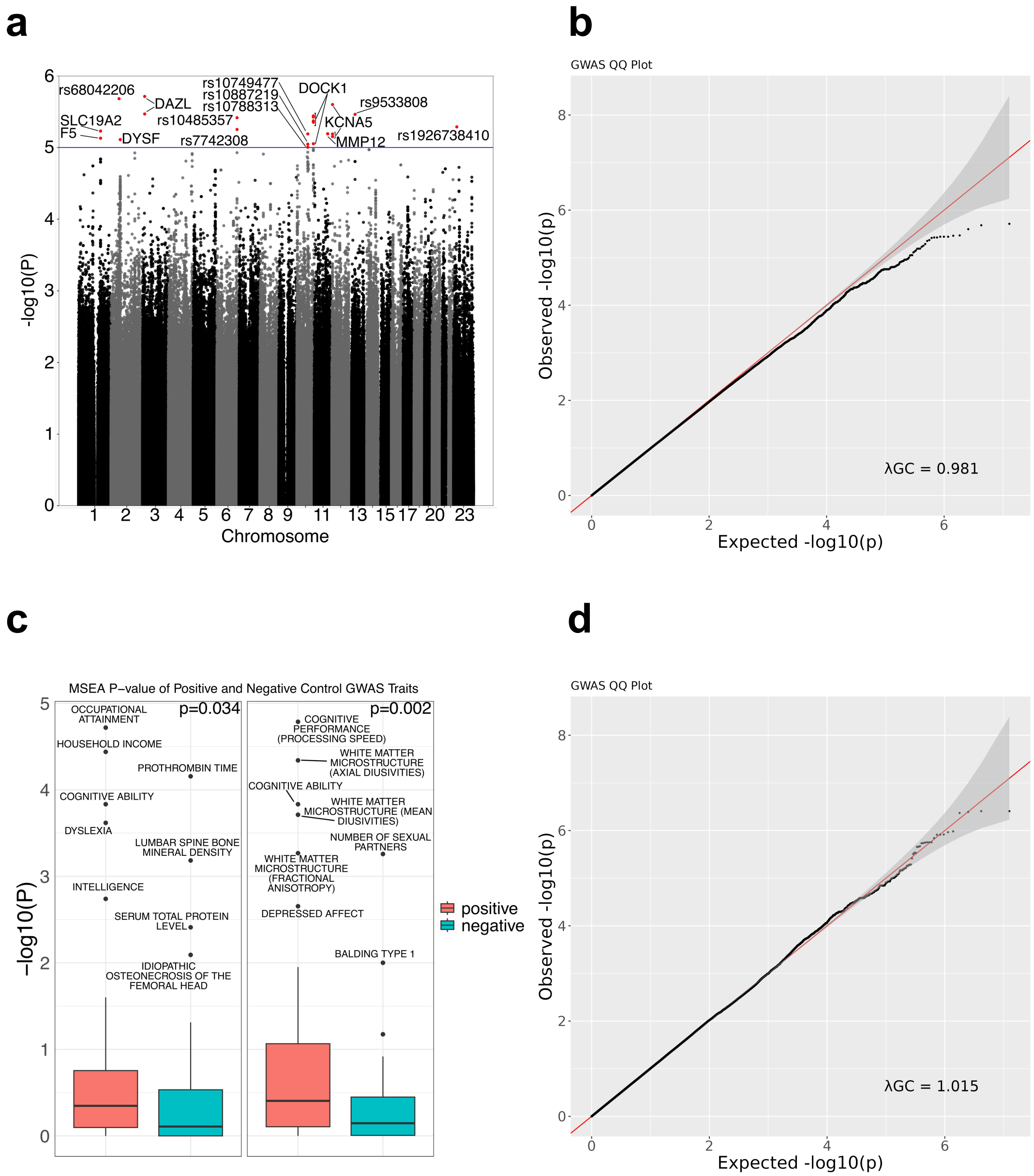

Supplementary Figure 2: Interaction GWAS summary statistics and validation with previous GWAS. a) Manhattan plot of the SNPxInjury interaction term. Blue line is the genome wide suggestive threshold p=1e-5. **b)** QQ-plot of GWAS interaction p-values. Genomic inflation factor of 0.981 indicates no systematic confounders, and the p value deflation is common for low sample sizes and over correction of genetic principal components. **c)** Distribution of MSEA -log10(p-values) in positive and negative control GWAS traits from GWAS catalog determined by 2 neuroscientists. Traits from each neuroscientist is displayed separately. The SNP-by-mTBI interaction signals show stronger enrichment for GWAS SNPs of positive control traits as determined by MSEA. **d)** QQ-plot of European-only SNP-by-mTBI interaction GWAS. Genomic inflation factor of 1.015 indicates no systematic confounders, and minimal p-value deflation is expected given the absence of multiple ancestry correction in a single-ancestry cohort.
